# On the use of deep learning and computational fluid dynamics for the estimation of uniform momentum source components of propellers

**DOI:** 10.1016/j.isci.2023.108297

**Published:** 2023-10-27

**Authors:** Raúl Martínez-Cuenca, Jaume Luis-Gómez, Sergio Iserte, Sergio Chiva

**Affiliations:** 1 Department of Mechanical Engineering and Construction, Universitat Jaume I, 12071 Castelló de la Plana, Comunitat Valenciana, Spain; 2 Department of Computer Science, Barcelona Supercomputing Center - Centro Nacional de Supercomputación (BSC-CNS), 08034 Barcelona, Cataluña, Spain

**Keywords:** Artificial intelligence, Industrial engineering

## Abstract

This article proposes a novel method based on Deep Learning for the resolution of uniform momentum source terms in the Reynolds-Averaged Navier-Stokes equations. These source terms can represent several industrial devices (propellers, wind turbines, and so forth) in Computational Fluid Dynamics simulations. Current simulation methods require huge computational power, rely on strong assumptions or need additional information about the device that is being simulated. In this first approach to the new method, a Deep Learning system is trained with hundreds of Computational Fluid Dynamics simulations with uniform momemtum sources so that it can compute the one representing a given propeller from a reduced set of flow velocity measurements near it. Results show an overall relative error below the 5% for momentum sources for uniform sources and a moderate error when describing real propellers. This work will allow to simulate more accurately industrial devices with less computational cost.

## Highlights

• Propellers modeling as momentum sources is essential for industrial tanks design
• A novel method to estimate source terms from velocity measurements was proposed
• The method combines the power of Computational Fluid Dynamics and Neural Networks
• Errors below 5% were found when inferring homogeneous momentum sources

## Graphical abstract

**Figure:**
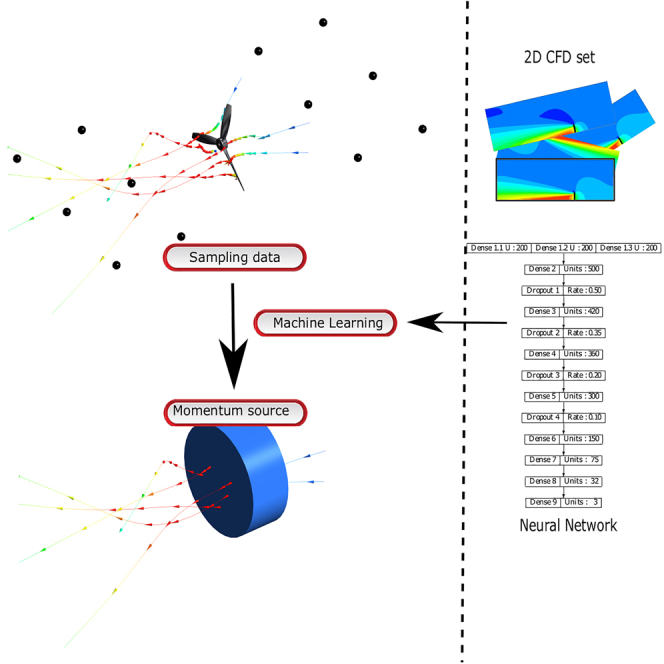

## Introduction

Most industrial applications basing on the treatment of fluids need for devices able to displace and/or mix the fluid^1^ such as pumps to activate the flow in pipelines, mechanical rotating mixers in the pharmaceutical industry, fans in ventilation, and so forth. Propellers are widely used for flow activation in various applications, including tanks, reservoirs, and marine environments. They are extensively used in aeronautics for the propulsion of ships, submarines, and aircrafts. In wastewater treatment industry,^2^ they activate the flow in biological reactors permitting the pass of the flow through alternating aerated and non-aerated regions. Propellers are also used in anaerobic digesters, where they serve to avoid dead volume formation in dynomix-based systems.^3^

The selection, placement, and orientation of the suitable propeller for a given application is a cumbersome task that relies on the knowledge gathered from previous experiences.^1^ But nowadays, it is possible to further improve the traditional designs thanks to the use of Computational Fluid Dynamics (CFD) simulations. In this way, CFD codes provide a virtual laboratory where it is possible to inspect the flow characteristics corresponding to different propeller configurations and propose modifications before the actual propeller implementation. This approach has been proven to be successful in fields such as aerogenerator design,^4^ wastewater treatment,^5^^,^^6^^,^^7^ or industrial mixing.^8^^,^^9^

At present, there are several frameworks available to include propellers in CFD simulations. The Dynamic Mesh (DM) framework provides the most realistic simulation approach as it introduces the full blades geometry moving in the domain.^10^ In this way, it is possible to resolve the complex transient vortex patterns that form in the vicinity of the blades. Given the geometry movement, the domain must be meshed every time-step thus resulting in a huge increase of the computational cost.^11^ To avoid the re-meshing procedure, Murthy et al. proposed the Sliding Mesh (SM) technique.^12^ Here, the domain is split into two regions that are meshed independently: a cylindrical region, named as disk, containing the blades geometry, and the rest of the domain, the so-called far-field. The disk mesh rotates around its axis during the resolution so the nodes in its frontier slide over the nodes of the static far-field mesh. The two regions are implicitly coupled at the interface separating the two blocks via a sliding-grid algorithm, which takes into account the relative motion between the two sub-domains and performs the required interpolations. Consequently, the SM also accounts for the local transient effects of the moving blades but at a reduced computational cost.^13^^,^^14^

The Multiple Reference Frame (MRF) was proposed to further reduce the computational cost of propeller simulations.^15^ As in the SM, the domain is divided into the disk and the far-field regions. But now there is no mesh movement, so the two regions can share the same conformal mesh. Blade movement is modeled by introducing a non-inertial rotational frame of reference limited to the disk mesh. These results in the apparition of additional source terms for the Coriolis forces in the momentum equations. Mesh nodes in the frontier between the two regions need special treatment to account for the two reference frames.^16^

Finally, the Momentum Source (MS) framework aims at providing a good description of propellers performance at a minimal computational cost. Here, the propeller's geometry is replaced by source terms in the momentum conservation equations.^17^^,^^18^ These source terms act within the disk region, providing a good description of the flow in the far field, i.e., far from the blades.^6^^,^^14^^,^^19^^,^^20^ Nevertheless, flow characteristics close to the blades tend to deviate from the actual values, specially in terms of transient velocity gradients and turbulence. Even though a transient version of the MS was recently proposed,^21^^,^^22^ the steady state version is of common use nowadays. At present, the main drawback of this technique is the determination of the proper momentum source components that characterize a given propeller. The mechanistic models for the calculation of the momentum are usually complex and require detailed knowledge of the blades geometry. In addition, these mechanistic approaches rely on rough approximations on the flow linearity thus limiting their application. As a result, propellers are modeled as momentum sources with just one axial component for practical applications, and it is mostly used for the simulation of big-sized tanks, specially in simulations regarding wastewater treatment.^23^^,^^24^^,^^25^

The new idea introduced in this article regarding the CFD simulation of flows involving propellers or other rotatory equipment is to use the MS model with a Deep Learning-based tool to accurately propose the momentum source components that better reproduce the averaged effects of the propeller. The proposed method can be extended in future to include more complex geometries, transient effects, non-uniform momentum sources, cross-flow or the presence of near walls.

The use of DL techniques is not new into the field of fluid mechanics, as they have been widely used to learn the mechanics of fluid flows.^26^ Particularly, Tamaddon-Jahromi et al.^27^ used DL to solve different inverse problems for heat transfer problems, obtaining the temperature boundary conditions from random measurements of the domain. Further deepening into inverse problems and its variants, Wang et al.^28^ managed to obtain the statistic expansion parameters for the hydraulic conductivity fields from direct random measurements of the hydraulic head field. In the field of marine propellers, Miglinti et al.^29^ proposed a hybrid method combining physical models and date driven models to predict the cavitation noise spectra produced by real propellers. Regarding the use of a virtual laboratory to obtain the results used for training an Artificial Neural Network, Calcagni et al.^30^ used computational models (Boundary Element Method) to numerically simulate the propeller's performance and use the hydrodynamics quantities obtained to feed their model. Using the aforementioned idea, Miglianti^31^ expanded these previous works by incorporating the Boundary Element Method to the workflow.

As for the resolution of inverse problems using Artificial Intelligence, this topic is becoming so popular that NVIDIA has developed SimNet (https://developer.nvidia.com/simnet), an accelerated simulation toolkit based on a physics-informed neural network (PINNs).^32^ The performance of SimNet has been demonstrated through the resolution of an inverse problem on a transient flow past a 2D cylinder.^33^

The innovative contribution of this work lies in proposing a new method to obtain uniform momentum source terms using the MS model as a starting point. Unlike traditional mechanistic approaches, this method is based on a hybrid workflow that combines CFD simulations and AI. In short, a set of 500 CFD simulations (400 to train, and 100 to test/validate) with different uniform momentum source terms serves to feed a predictive model based on AI. The resulting predictive model provides a momentum source with uniform components from a set of velocity measurements in order to enable the CFD calculation of flow fields similar to the experimental ones.

Next sections are devoted to introduce the proposed method and demonstrate its feasibility using the virtual scenarios provided by CFD simulations. First, results shows the results of the proposed approach demonstrating its validity. Later, discussion presents the main insights of the method and discuses about the obtained results and future work to improve and expand the novel idea explored in this article. To provide more information about the reproducibility of the presented work, STAR Methods section introduces the basics of the CFD formalism as well as the momentum source approach for the simulation of propellers. Also explaining the full methodology to solve the inverse problem, from the full CFD configuration to the procedure for the training and evaluation of the DL predictive model.

## Results

This section focuses on analyzing the quality of the NN predictions. To this purpose, the 100 CFD cases of the validating subgroup served as as virtual laboratory. In this way, the corresponding velocity samples will be noted as “experimental” values. The NN served to obtain the predicted momentum components from the velocity fields of the testing subgroup. The actual momentum values will be noted as M, whereas the corresponding values predicted by the NN will be noted as $M^{\prime }$. Later, a new set of CFD cases were launched using the 100 predicted momentum sources. The resulting velocity samples at 200 points (20 radial and 10 axial, see Figure 4), $U^{\prime }$, will be compared to the “experimental” ones, U, to illustrate the performance of the proposed tool as a method to obtain the momentum source that generates a given experimental velocity field. Finally, results from the simulation of the real propeller will be compared with those obtained from the momentum source approach with the momentum sources proposed by the model.

### Comparison between model's predictions and original data

**Figure 1:**
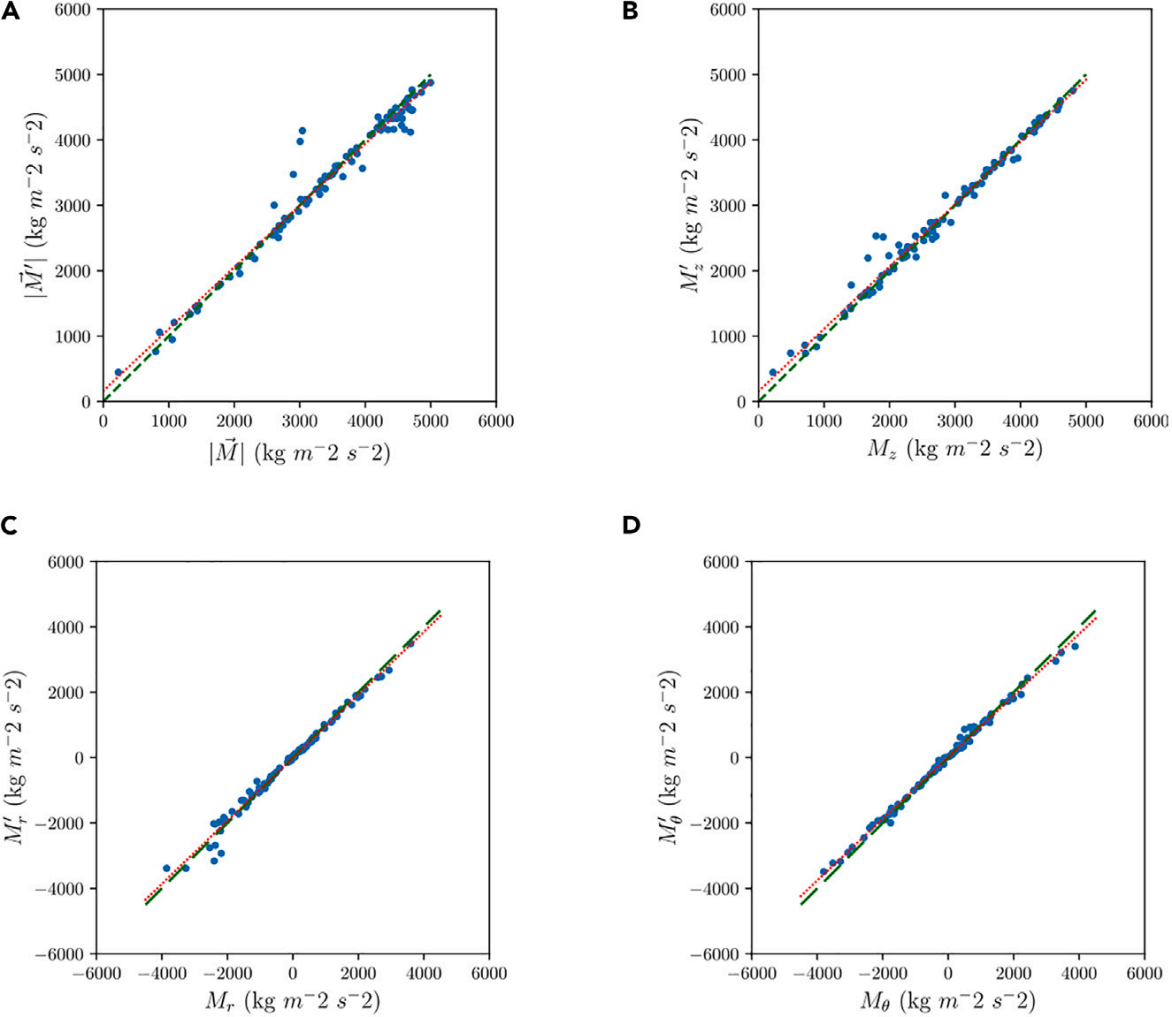
Comparison between predicted and actual momentum for the virtual experiment (A) Modulus. (B) Axial component. (C) Radial component. (D) Azimuthal component.

The results from this virtual experiment are illustrated in Figure 1. Figure 1A compares the predicted values for the momentum moduli, $M^{\prime }$, to the actual ones, *M*. From this figure, there is an apparent correlation between predicted and actual momentum modulus. The remaining plots show a similar degree of correlation for the momentum source components. In the Figure 1, the dots are the points where each prediction meets the real momentum term value, while the dashed green line is a zero error line of slope equal to unity and the dotted red line is the result of the linear regression. The properties of the four linear correlations (in the form $f^{\prime }=mf+n$) are summarized in Table 1 to provide a quantitative evaluation of the NN performance in terms of slope, *m* (should be close to 1), intersect, *n* (should be close to 0), and coefficient of determination ($R^{2}$, should be close to 1).

**Table 1:** Correlation data for the plots in the comparison between predicted and actual momentum for the virtual experiment

	m[-]	n [kg m^2^ s ^−2^]	R^2^ [-]
M^’^	0.964	−9.28	0.985
M_z_^’^	0.943	8.76	0.995
M_r_^’^	0.953	157.08	0.983
M_t_^’^	0.944	164.61	0.967

### Comparison between the predictive model and other shallow algorithms

Previous section demonstrated that an NN can serve to provide accurate predicitons when dealing with uniform momentum sources. However, one might think that the same task could be performed by using shallow algorithms.

**Figure 2:**
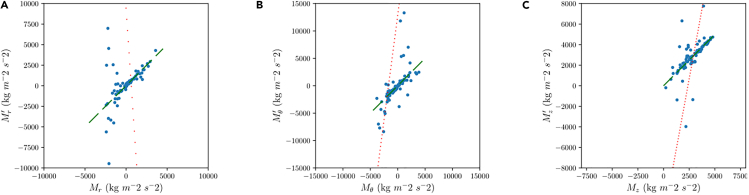
Comparison between predicted and actual momentum for the multivariate linear regression model (A) Radial component. (B) Azimuthal component. (C) Axial component.

First, a Multiple Linear Regression (MLR) was tested using the 400 training CFD cases. The MLR was implemented taking advantage of the *LinearRegression* class of the module *scikit-learn*. The model used the same number of inputs than the NN model. Figure 2 shows the same comparison as Figure 1, comparing the predicted momentum terms by the linear regression with the real values. Although one can note a certain linear trend, there is a significant amount of outliers that make this approach unfeasible. The slopes and coefficients of determination for the three momentum components in Table 2 further demonstrate that MLR fails to obtain satisfactory results.

**Table 2:** Correlation data for the plots in Figure 2

	m[-]	R^2^ [-]
M_z_^’^	−14.81	0.135
M_r_^’^	7.64	0.01
M_t_^’^	5.44	0.004

**Figure 3:**
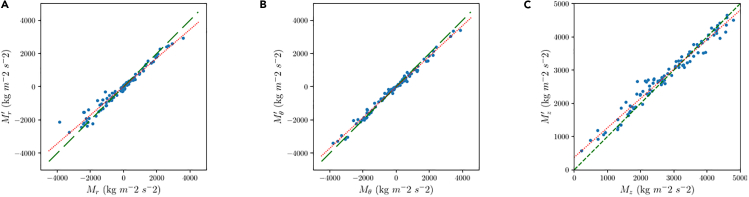
Comparison between predicted and actual momentum for the random forest model (A) Radial component. (B) Azimuthal component. (C) Axial component.

Second, a test was made using a Random Forest (RF) approach, configuring the number of inputs to use the same as the MLR and NN models. This model was created from the class *Random Forest Regressor* from the module *scikit-learn*. The other configurable parameters were set to the default values. A test was performed using different depths of the forest, finding that there was no difference between using a depth of 5 and 10. For the RF of depth 10, the obtained results are shown in Figure 3.

From Figure 3, it can be seen that in all the momentum components, the predictions have better behavior that the MLR model. In the view of Table 3, one can note that slopes and coefficients of determination are better than for the MLR, but still fall far behind from those obtained with the NN.

**Table 3:** Correlation data for the plots in Figure 3

	m[-]	R^2^ [-]
M_z_^’^	0.859	0.964
M_r_^’^	0.923	0.991
M_t_^’^	0.855	0.969

### Comparison between “experimental” and predicted velocity fields

The strong correlation between momentum components when using the NN is inherited by the velocity samples. This section compares ”experimental” velocity samples with predicted ones, thus allowing to check the performance of the NN from the point of view of the users. Table 4 shows the correlation data for velocity moduli and components. As a final quantification of the error in the proposed approach, we propose to use the following error metric:

(Equation 1) $$\epsilon_{i,j}=\frac{1}{N_{s}}\frac{\sum_{n=1}^{N_{s}}abs\left(U_{i,j,n}^{\prime }-U_{i,j,n}\right)}{abs\left(\Delta U_{i,j}\right)}\text{,}$$

where $\Delta U_{i,j}$ is the range of values for the i−th component of the velocity studied in the j−th case, i.e., $\Delta U_{i,j}=\max\left(U_{i,j,n}\right)-\min\left(U_{i,j,n}\right)$ . This metric was chosen as it is similar to the normalized residual used by the ANSYS CFX solver to estimate the convergence error of the iterative CFD resolution.^34^ Accordingly, 95% of the cases have an error below the 5% in the velocity components. It is worth to mention that the mean error for the axial component was 1.03%, 2.68% for the radial component, and 3.21% for the azimuthal one.

**Table 4:** Correlation data for the velocity fields

	m[-]	n [m s ^−1^]	R^2^ [-]
U^’^	0.964	0.0019	0.94
U_z_^’^	0.964	−0.0017	0.932
U_r_^’^	0.995	0.0034	0.980
U_t_^’^	0.992	0.002	0.982

**Figure 4:**
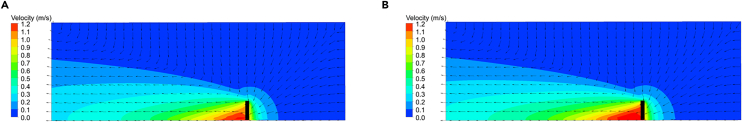
Contour maps for velocity fields (A) The "experimental" case. (B) The corresponding CFD calculation.

This strong agreement becomes apparent in Figure 4. The figure on the left side shows the “experimental” velocity field for one of the 100 cases, whereas the figure on the right side shows the corresponding predicted velocity field. Note that both fields are almost indistinguishable.

**Figure 5:**
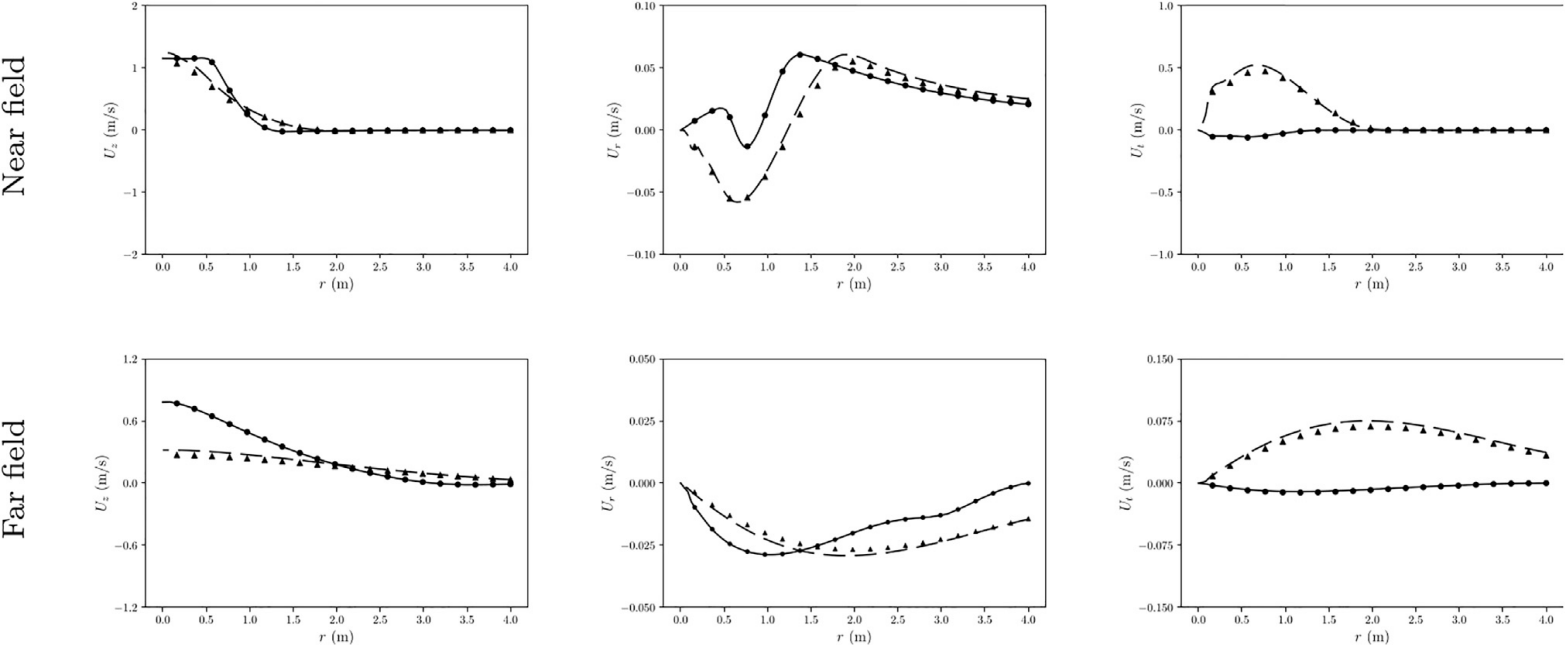
Velocity component profiles for two cases, one with low error (continuous line) and one with high error (dashed line) according to the proposed error metric Lines represent original CFD data and markers CFD results with the corresponding predicted momentums.

To further illustrate the performance of the proposed methodology, Figure 5 shows radial velocity profiles for two cases. The profiles drawn with a solid line correspond to a case with a relative error in axial component of 0.03% according to the metric in (1). Note that the profiles match so perfectly that “experimental” and predicted profiles overlap. Dashed lines correspond to case with an error of 2.15% (93% of the cases have an error below this case). Here, the predicted profiles slightly deviate from the “experimental” ones but the overall trends are captured quite nicely. Note that. for each case, “experimental” and predicted profiles of the three velocity components have been represented at the two lines of 4m of length in the radial direction (at distances of 1m and 10m after the actuator disk).

### Comparative results with CFD real propeller simulation

A full CFD transient simulation of a complex propeller was performed. To obtain the momentum source components, the resulting velocity fields where spatially averaged at a given time-step (across the azimuthal direction) to obtain 2D averaged velocity fields. The results were similar to those obtained after a time-average of the velocity fields over 3 rotations of the propeller.

**Figure 6:**
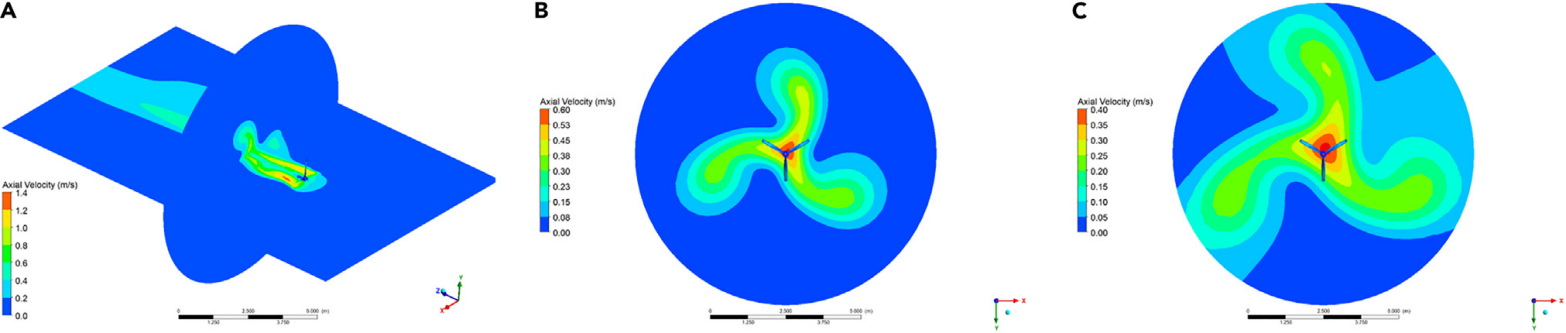
Velocity contours for the XZ plane at different distances from the propeller (A) z = 2.5 m. (B) z = 5 m. (C) z = 7.5 m.

Figure 6 represents the simulation at a particular time-step displaying velocity contours at different distances from the propeller. The effect of the rotation on the flow generates visible patterns of swirling zones in the domain which continue to rotate as they travel away from the propeller. These zones justify the averaging process in order to obtain a representative steady-state velocity field.

**Figure 7:**
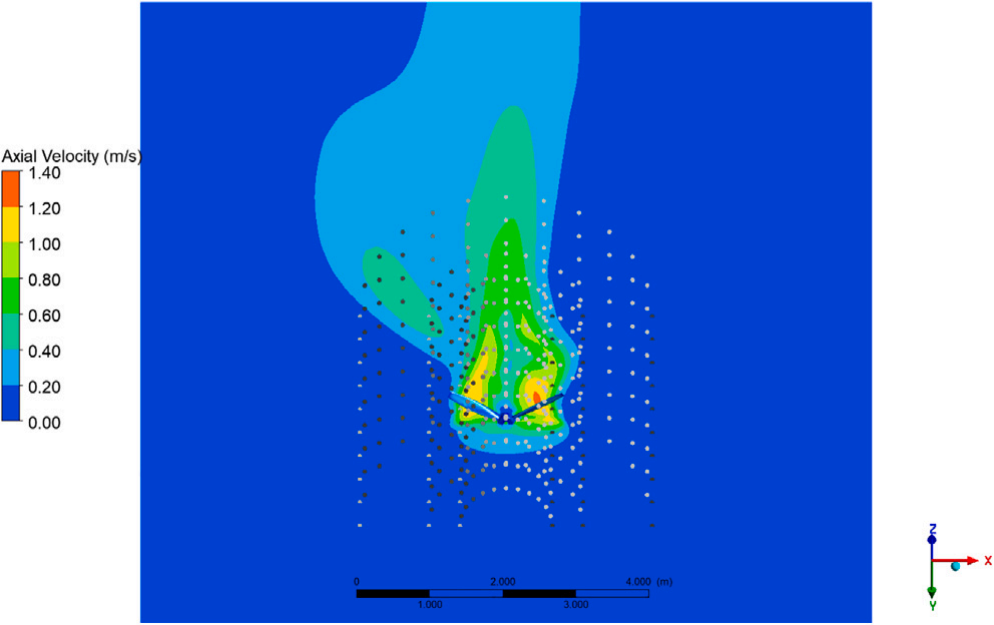
Example of three averaging cylindrical samples at different radial distances from the propeller In this illustration, each cylinder contains 10 circumferences, with 25 points along a complete revolution.

For the averaging process, 20 cylindrical surfaces of revolution were created (with different radii), each one with 10 circumferences (at different axial locations) of points to obtain the original 20x10 (radial, axial points) input space that feeds the NN. Each circumference contains 360 points, one per angle of rotation. Figure 7 shows an example of three cylinders at different radial distances.

Next, the averaged data was normalized following the same process as the one performed to the initial dataset, and momentum predictions were generated with the NN. The value of them were as follows: $M_{r}=-114.5kgm^{-2}s^{-2}$, $M_{t}=-1008.4kgm^{-2}s^{-2}$, and $M_{z}=1321.0kgm^{-2}s^{-2}$. The predictions were then used in an MS simulation with the same setup as the ones generated as the dataset.

**Figure 8:**
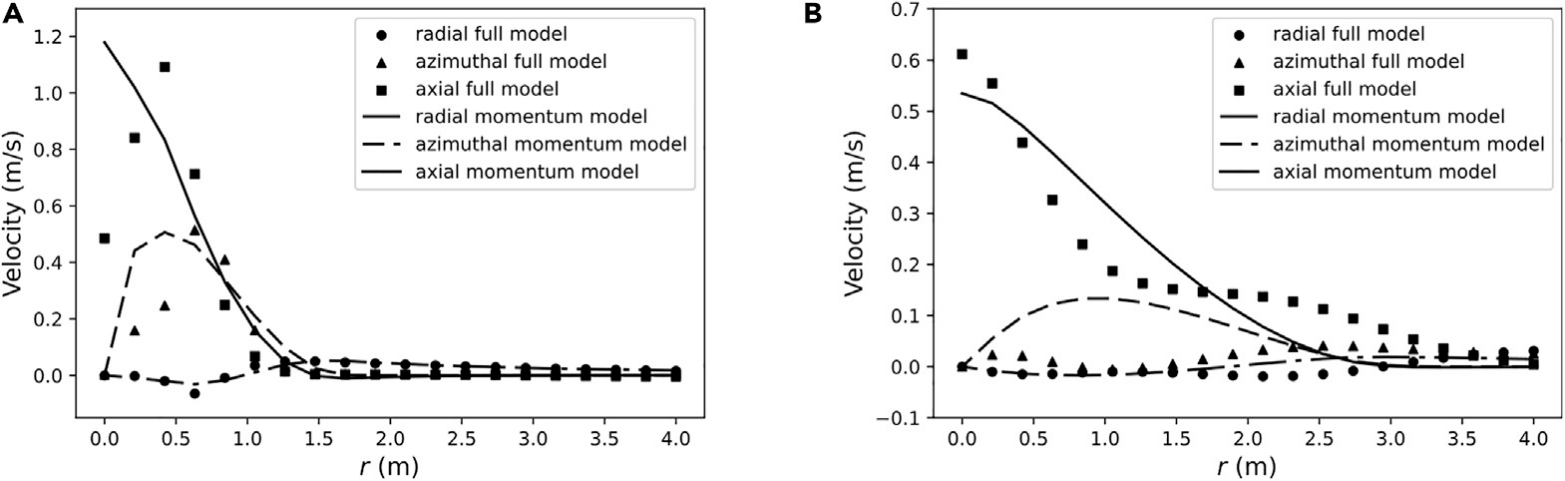
Velocity component profiles at different distances from the propeller, comparing the data obtained from the simulation of a real propeller with the MS simulation of the NN’s predictions. (A) z=1 m. (B) z=5 m.

Finally, the velocity profiles were extracted at 1 and 5 m from the propeller, as done in the previous subsection. Figure 8 shows that the tendencies were mostly captured with some differences specially located near the axis of the domain. The most noticeable is the decay of the axial velocity near the center, while the momentum simulations predict a maximum on the axis for this component. This minimum near the axis can be understood as the blades of the propeller move at a really slow velocity near the axis. Thus, right in the location where the propeller is not generating any thrust the momentum source is adding thrust.

In addition, the section of the blades is not constant along the entire radial length and their speed increases linearly with the radial distance, which causes variation in the drag and lift forces exerted on the fluid. This non-uniform distribution of the forces indicates that a non-uniform momentum source should be utilized. However, the neural network used in this study was trained on simulations with uniform momentum sources. As a result, the NN is not able to provide a full prediction of the momentum source. This explains the main differences in the profiles shown in Figure 8.

## Discussion

The CFD modeling of propellers needs for a reliable methodology to speed up the simulations while keeping the quality of their results. The momentum source approach aims at this purpose as it permits a significant reduction of the computational cost. However, the quality of the resulting simulations with the methods available up to date needs to be improved.

This article aims at introducing a new method to obtain the momentum source components of propellers from experimental velocity values thanks to the advantages of DL. The method consists in the training of an NN from a set of CFD simulations comprising momentum sources with a random distribution of its component values. The resulting NN is able to estimate the momentum source parameters from a set of velocity measurements.

The feasibility of this novel approach was demonstrated in a virtual environment that takes the profit of CFD simulations to provide the velocity distributions caused by a set of 100 propellers with momentum components (below $5000kgm^{-2}s^{-2}$). For every case, a set of 200 “experimental” velocity samples were passed to the NN to estimate the momentum source components. The overall performance was quite good as the relative error between the velocities provided by the simulation and the “experimental” velocity samples remained below 5% for 95% of the cases.

From this first part, two conclusions can be made: first, the resulting predictive model is able to estimate the momentum source parameters from a set of velocity measures (the training process was successful). And second, the fast learning of the model indicates that the available information during the training was big enough. Further work can be done in order to study the possibility of reducing the number of inputs, i.e., the number of velocity samples while keeping the error committed as low as possible. In this sense, an input sensitivity study is intended to be made in the future.

To determine whether the use of a Neural Network (NN) was necessary, we tested two simpler predictive models: Multiple Linear Regression (MLR) and Random Forest (RF). The results obtained from the MLR indicated that such a simple model cannot effectively capture the non-linearities of the inverse problem. Although the RF produced results close to those of the NN and showed promise, future developments of this work aim to predict non-uniform momentum and turbulence sources, which may require more complex and deep algorithms such as an Artificial Neural Network. Thus, a predictive model based on an NN was chosen for further study in this article.

Although existing some discrepancies regarding the momentum prediction compared with the actual momentum terms for the NN predictive model, the third part of the results show that there exists a general agreement in the sampled velocity points shown in Figure 5. For the cases with bigger errors, the profiles far from the source show acceptable agreement. Since the MS approach is used to capture general characteristics of the flow, specially at the far field, poor predictions could still be better than experience based momentum sources in cases where no geometry information about the propeller is available.

With respect to the last validation test, discrepancies were observed when comparing the MS simulation with the averaged results from the SM. Some of these discrepancies were attributed to the central axis in the geometry that holds the blades, which does not add momentum to the fluid in that zone. In contrast, in the MS simulation, the momentum source reaches the center of the domain, where the propeller is not generating thrust, resulting in slower velocities near the axis. However, the momentum source is still adding thrust, which explains the differences observed between the two simulations.

Furthermore, it should be noted that the section of the blades is not constant along the entire radial length and their speed increases linearly with the radial distance, which causes variation in the drag and lift forces exerted on the fluid. This non-uniform distribution of the forces indicates that a non-uniform momentum source should be utilized. However, the neural network used in this study was trained on simulations with uniform momentum sources, and therefore, it is not intended for non-uniform inferences since it has not been trained for that purpose. This limitation explains the main differences in the profiles shown in Figure 8. While the NN provides a good-enough result for must purposes, note that it serves as a proof of concept for this study. Further development is necessary to improve the results for complex propellers.

Despite the promising outcomes achieved in the first part of the results section, further development of the proposed method is required in the future. Although the presented results show that the proposed approach is feasible for accelerating CFD simulations of propellers and provides acceptable momentum sources, the validation with the simulation of a real propeller highlights the necessity of conducting additional research to achieve higher and more robust applicability. To overcome this limitation, future work is needed to introduce non-uniform momentum sources, turbulence sources, the effects of cross-flow, and so forth. Moreover, it is of great interest to check its performance in real scenarios, measuring the velocity fields produced by actual propellers in mixing tanks. In this direction, the reduction of the required number of inputs (velocity measurements) will facilitate the experimental tasks. These aspects are beyond the scope of this article, that focuses on the proposal of the new methodology for the estimation of the momentum source components of propellers.

### Limitations of the study

This study has been made to develop a new method for the estimation of uniform momentum source components of propellers in a controlled environment such as a virtual laboratory with fluid simulations. Thus, it has not been tested using real propellers nor real velocity data, nor its predictions regarding non-uniform forces from real propellers will yield results as promising as for uniform momentum forces. The momentum sources are limited to a maximum module of $5000kgm^{-2}s^{-2}$.

## STAR★Methods

### Key resources table

**Table:** 

REAGENT or RESOURCE	SOURCE	IDENTIFIER
**Deposited data**		

Data set for training	This paper	https://github.com/JaumeLG/MomentumSourceNNInverseModel/
Data set for validating	This paper	https://github.com/JaumeLG/MomentumSourceNNInverseModel/

**Software and algorithms**		

ANSYS-CFX 20.2	ANSYS simulation software	https://www.ansys.com/
Anaconda	Anaconda	https://www.anaconda.com/
Jupyter Notebook	Jupyter Hub	https://jupyter.org/
Python version 2.8.2	Python Software Foundation	https://www.python.org
Matplotlib version 3.6.0	Matplotlib	https://matplotlib.org/
Keras version 2.7.0	Keras SIG	https://keras.io/
Numpy version 1.21.4	NumPy	https://numpy.org/
Pandas version 1.3.4	NumFOCUS	https://pandas.pydata.org/
Scikit-learn version1.0.1	Scikit-Learn	https://scikit-learn.org/stable/
Tensorflow version 2.7.0	TensorFlow	https://www.tensorflow.org/

### Resource availability

#### Lead contact

Further information and requests for resources should be directed to and will be fulfilled by the lead contact, Raúl Martínez Cuenca (rcuenca@uji.es).

#### Materials availability

This paper did not generate new unique materials.

#### Data and code availability

• To provide more information about the dataset, the processing of the data, the predictive model or the training process, a GitHub link (https://github.com/JaumeLG/MomentumSourceNNInverseModel) to a public repository is added. The data used to train the predictive model is contained in the GitHub repository as a zipped folder called *“Dataset”* with the corresponding 500 CSV files. The other 100 files obtained after simulating the NN's predictions from the testing subset used in the validation process are also provided in another zipped folder (*“ValidationData”*).
• A python file with the corresponding code used in the paper is supplied where the reader will be able to reproduce the trained model in the paper. All the code used in this paper has been created by the authors of the present study.
• Any additional information required to reanalyze the data reported in this paper is available from the lead contact upon request

### Method details

#### Momentum source approach: formalism

This subsection starts introducing the flow equations underlying the CFD simulations, pointing out the source terms that aim at representing the effects of propellers. This description is completed with a critical discussion on the use of mechanistic methods to obtain the momentum source of actual propellers. From this discussion, it is apparent the need of a more practical tool.

#### Conservation equations

At present, CFD simulations at industrial scales use the so-called Reynolds-Averaged Navier-Stokes (RANS) formalism. For isothermal incompressible flows, the flow dynamics is governed by the following equations.

• Continuity:

(Equation 2) $$\frac{\partial \rho }{\partial t}+\nabla \cdot \left(\rho U\right)=0\text{.}$$

• Momentum:

(Equation 3) $$\frac{\partial }{\partial t}\left(\rho U_{i}\right)+\nabla \cdot \left(\rho U_{i}U\right)=\nabla \cdot \left(\Gamma_{i}\nabla U_{i}\right)+S_{i}\text{.}$$

In these equations, $U_{i}$ stands for the flow velocity components, ρ the mean fluid density and $\Gamma_{i}$ the turbulent diffusivity. The term $S_{i}$ stands for the external momentum sources:

(Equation 4) $$S_{i}=-\frac{\partial P}{\partial x_{i}}+\rho g_{i}+M_{i}\text{,}$$

being *P* the mean pressure, $g_{i}$ the gravity vector components and $M_{i}$ the momentum source term. When flow turbulence is described with the k−ϵ model, $\Gamma_{i}=\mu +\mu_{\tau }$, with

(Equation 5) $$\mu_{\tau }=C_{\mu }\rho \frac{k^{2}}{\epsilon }\text{,}$$

and $C_{\mu }=0.09$. The two turbulence field variables, *k* and ϵ, are governed by their corresponding transport equations. More details on the transport equations and the involved constants can be found in the ANSYS-CFX Theory Guide.^34^

It should be noted that the existence of a momentum source should be physically linked to an extra turbulent kinetic energy source term and possibly a turbulent kinetic energy dissipation source term. However, in the bibliography there are no models for the momentum source approach that also include a source term for the turbulent kinetic energy. As in previous works, this study aims to obtain satisfactory momentum source terms to reproduce the velocity field.

The k−ϵ model is used because it is a computationally cheap model which provides good velocity profiles and it has been extensively used in the context of CFD simulation of big tanks, specially in the wastewater treatment industry. Also, since it is a steady-state simulation with average values and there are no wall effects, there is no need to use another turbulence model more accurate or with better resolution near the walls.

#### Momentum source approach

The MS approach was first applied to CFD simulations in the work of Rajagopalan and Fanucci.^17^ They proposed to introduce the effects of a straight-bladed Darrieus rotor on air flows through the terms $M_{i}$ in Equation 4. These terms acted just within a cylindrical region, named as actuator disk, with its axis coincident with the rotor axis and a radius similar to the blade length. The disk height was set to the maximum axial extent of the blades. To calculate the momentum source terms, they first calculated the total force of the blades on the flow over one rotating cycle. To do so, they considered each blade as a succession of infinitesimal aerofoils across the radial direction, *r*. Then, these forces should be averaged over one rotating cycle taking into account the number of blades and their angular extent. Finally, the momemtum source components were calculated as the force per unit volume.

This mechanistic approach has two main drawbacks. First, its application to blades with complex geometries requires the identification of the individual airfoil sections, so the actual geometry of the propeller blades must be perfectly known.^35^ At present, this information is not provided by manufacturers, so the users must find a proper method for the 3D characterization of the shapes and get their drag and lift coefficients. Second, the direct integration of forces assumes that the flow dynamics is local and that its effects are linear, which of course is not generally the case.

Other mechanistic approaches also rely on flow assumptions that lack of general validity^35^ and the techniques involved are usually difficult to apply, specially when dealing with propellers that have been already installed in the facilities. These mentioned assumptions usually involve: generation of simple 2D eddies, absence of a tip-blade factor to account for the effect of lift reduction due to vortex shedding, use of lookup tables for drag and lift coefficients or even specific Reynolds-depending functions for those coefficients.

As a result, recent engineering CFD studies tend to oversimplify the implementation of the momentum source. Indeed, the most practical approach is to assign a uniform value to the axial component of the momentum source. Then, the momentum is calculated from the thrust force. Finally, the efficiency of the propeller is tuned so that the resulting CFD velocities (or flowrate) match experimental data. This may be useful for applications involving tanks of big dimensions, such as those used in wastewater treatment.^6^^,^^24^^,^^25^ But some applications, specially in small tanks, require a most accurate description of the propellers momentum source. In any case, a practical tool for the calculation of the momentum components of propellers would imply a significant advance in their CFD modeling.

#### Methodology

This subsection provides a detailed description of the new method proposed for the estimation of the momentum source components of a given propeller from a set of velocity measurements. First step consists in the CFD computation of the flow generated by a an actuator disk in open waters. This computation is performed for a set of *J* known momentum sources, $M=\left\{M_{1},M_{2},\ldots ,M_{J}\right\}$, that differ in their component values. Next, the resulting set composed by *J* momentum sources and the corresponding *J* velocity fields serve to train and validate a Neural Network (NN). The proposed NN takes as input a set of samples of the velocity field caused by a given propeller and provides as output an estimation (or prediction) of the corresponding momentum source components, $M_{i}^{\prime }$ .

#### CFD training set

Since this paper pretends to develop a novel tool for the MS model, the simulations used in this work are designed to be as simple as possible to test the tool and not be influenced by possible numerical errors of the simulations. Also, it has been intended to emulate the working conditions of the propeller in a big-sized tank such as in the wastewater treatment industry, a field where the group has previous experience performing simulations.

Therefore, the propeller is assumed to be located in open waters, i.e., far from walls and obstacles to prevent their influence on the resulting flow. As depicted in Figure 1 of the supplemental information, the flow domain is restricted to a $10^{\circ }$ slice of a cylinder with a radius of 5m and a length of 15m. The propeller geometry is replaced by an actuator disk with a radius of 1m and an axial extent of 0.2m that is radially centered with the domain but axially displaced 5m respect one of the sides. This simple geometry allowed for the generation of the conformal-structured mesh shown in Figure 1 of the supplemental information). The momentum source is subdivided into cells that spread uniformly in each direction: 15 radial, 10 axial, and 10 azimuthal. The 4.9m between the momentum source and the “inlet” to the domain are axially resolved with 40 cells non-uniformly spread (a bias factor of 20 toward the disk). Similarly, the 9.9m between the source and the “outlet” are resolved with 90 cells (bias factor of 15 toward the disk). The 4m of radial shell between the actuator disk and the domain borders is split into 25 cells with a bias factor of 4 toward the disk. All the bias factors have been set so that the cells that are in contact on each side of the actuator disk limits have a similar size thus ensuring a smooth transition between the cells.

The simulation is configured so that all the domain boundaries (except for symmetries) are set as openings, i.e., flow can either enter and leave the domain (even at the so-called “inlet” and “outlet” surfaces). The domain is filled with water ($\rho =997\;kg\;m^{-3}$, μ=0.001Pa·s) and heat transfer is not considered, so an isothermal Newtonian incompressible flow is assumed. The momentum source was implemented in the cylindrical volume as

(Equation 6) $$M=M_{r}{\hat{u}}_{r}+M_{\theta }{\hat{u}}_{\theta }+M_{z}{\hat{u}}_{z}\text{,}$$

being ${\hat{u}}_{r}$, ${\hat{u}}_{\theta }$, and ${\hat{u}}_{z}$ the unit vectors in cylindrical coordinates. For every case, the momentum components are set as uniform, i.e., they do not change with the position inside the actuator disk.

ANSYS-CFX 20.2 was used to solve the flow equations. The iterative resolutions were performed using an adaptative time step to ensure a proper convergence. Accordingly, the first 100 iterations were performed with a time step of 2s. Then, it was reduced to 1s for the next 100 iterations. Finally, it was further reduced to a value of 0.2s until convergence at an RMS error of $10^{-5}$. The numerical system was solved by using the high-resolution advection scheme and first order turbulence numerics. Figure 2 of the supplemental information shows the resulting velocity profiles for three different momentum sources.

To train and validate the NN, a total of J=500 cases were generated by changing the three components of the momentum source within the actuator disk. The components were defined in cylindrical coordinates and were limited to the range $\left(0,5000\right)kgm^{-2}s^{-2}$ for the axial component, and to the range $\left(-5000,5000\right)kgm^{-2}s^{-2}$ for the remaining ones. The set of J momentum sources was created in block to respect two restrictions: first, the module of the vector defined by the three components must have a maximum value of $5000kgm^{-2}s^{-2}$; second, the axial component must represent at least one-half of the total module to provide flow propulsion toward the ”outlet” region. Thus:

(Equation 7) $$M_{z}>0.5\cdot \left|M\right|\text{.}$$

To ensure that there is an equalized distribution of cases through all the possible cases' domain, for each bin of $M_{z}$ values, the total amount of possible cases that fulfill both restrictions was calculated. From this amount, a proportional number of cases was created in this subdomain, and this procedure was repeated through the complete range of values for $M_{z}$.

The final set was composed by the J×3 momentum components and the corresponding velocity fields. To reduce the amount of data sent to the NN, the velocity fields were sampled at the 200 points shown as black dots in Figure 2 of the supplemental information to get a total of J×3×200 velocity values.

#### Neural network training and validation

Given the complexity and scale of the problem being studied, the predictive model developed in this work utilizes deep learning techniques. Specifically, this section provides a comprehensive description of the neural network design and the steps taken to configure and fine-tune it to minimize prediction errors. To demonstrate its effectiveness, the proposed model’s performance is be compared with that of various shallow algorithms. Through this comparison, it becomes evident that the proposed model exhibits superior performance.

The process of designing a neural network architecture typically involves a combination of domain knowledge, experimentation, and trial and error to identify the optimal solution for a given problem. In line with this approach, the input and output layer sizes for the neural network presented in this work were tailored to the specific characteristics of the problem being addressed. The nature of the problem was found to align well with the use of a multilayer perceptron (MLP) neural network architecture, which was constructed using the MLP class available within the Keras and TensorFlow modules.

Given the lack of *a priori* evidence suggesting that certain inputs, in terms of spatial position or velocity components, are more crucial than others in determining the final output value, we opted to use fully connected layers, as is the case in the MLP class. By using this approach, each input provides its information to every initial neuron in the first layer, thus enabling all inputs to contribute to the final output. This strategy allows the learning process to determine the optimal weights for each input, thereby facilitating the identification of the most critical features for accurate prediction.

To achieve the desired predictive accuracy and address the overfitting problem, we designed a neural network that incorporates both hidden and dropout layers. The decision to include dropout layers was made during the experimentation phase, when different architecture variants were tested to reduce the overfitting observed during training. Given that the dimension of the input domain was two orders of magnitude greater than the output dimension, we chose to use an input layer with the same number of neurons as the total inputs. From there, we gradually reduced the number of neurons through each subsequent layer until we arrived at a final layer with the size of the output dimension. By taking this approach, we aimed to minimize the complexity of the model while retaining the predictive power necessary to accurately predict the desired outcome. Table 1 of the supplemental information illustrates the different layers of the neural network with the number of neurons per layer.

To optimize the performance of our neural network, we carefully selected the number of hidden layers, the activation functions, and the optimization algorithm based on their ability to minimize the loss function that best fits the model. Additionally, we made adjustments to other key parameters such as the size of the training, validation, and evaluation datasets, the mini-batch size, and the number of epochs to accelerate the training process while also minimizing prediction error and preventing overfitting. Through a process of fine-tuning and optimization, we systematically modified the architecture and training process of the neural network to improve its overall performance on the problem at hand.

About the previously mentioned Table 1 of the supplemental information, the input layer is composed of 600 neurons (three velocity components for each of the 100 sampling points), while the output layer is formed of three neurons (the three predicted momentum components). To enhance the performance of the predictive model, the first layer was divided into three parallel layers, each one receiving 200 input data corresponding to the velocity samples in each spatial coordinate. This component sorting prevents the information to be excessively mixed and obstruct the training process. Thus, the model has into account separately each velocity component to help the predictions. In addition, seven hidden layers were added with different neuron amount to minimize the training error and obtain better performance. To complement the hidden layers, three dropout layers where added to reduce the chances of appearing overfitting. Each dropout layer was given a dropout rate (percentage of the neurons whose weight must be set to zero) as shown in Table 1 of the supplemental information.

Given the non-linearity nature of the problem to solve, non-linear activation functions were used together with the neurons in the neural network. Particularly, this activation function was the LeakyReLU (Leaky Rectified Linear Unit) function, with a value for the alpha parameter of 0.2. An additional training was performed using the ELU (Exponential Linear Unit) function,^36^ however, an outperforming was observed when the LeakyReLU function was used, so it stayed as final choice.

As for the hyper-parameters selection process, several training tests were conducted involving: alpha parameter in LeakyReLU or ELU activation function; dropout rate of each dropout layer; learning rate; optimizer used; batch size and other possible hyper-parameters. The tunning of these hyper-parameters was always accompanied by an evaluation of the predictive model. The final selection of their values was made observing the metrics' values after running an evaluation of the trained NN using the Keras module function “evaluate” on the testing subset.

In most cases, error estimation was conducted with the mentioned Keras function (focusing on the loss metric described in Equation 7). In addition to evaluating the metrics on the testing subset, the training and validation errors were also analyzed during the training process. By monitoring the evolution of these curves, the occurrence of overfitting was detected, aiding in the adjustment of the predictive model’s hyperparameters. The hyperparameters were adjusted until the minimum error was achieved, at which point they were considered optimal and fixed.

At the end of each iteration, the network calculates the difference between the expected result and the prediction given by the internal calculation using its internal parameters. This difference is computed using the mean square error loss function:

(Equation 8) $$MSE=\frac{1}{3\times J}\sum_{i=1}^{3}\sum_{j=1}^{J}{\left(M_{j,i}-M_{j,i}^{\prime }\right)}^{2}\text{,}$$

where $M_{j,i}$ is the i-th component for the j-th known momentum source, and $M_{j,i}^{\prime }$ i-th component for the corresponding momentum source predicted by the NN.

Since the objective is to minimize this error, the back-propagation algorithm adjusts the weights and biases of the connections within the neural network computing the gradients which point how those parameters need to be changed to minimize the error. For each epoch, the neural network calculates the loss value of the mini-batches in which the training dataset is divided and updates the values of the network parameters to reduce the error in the next iteration. To do so, the *ADAM* optimizer^37^ was used as back-propagation method. The learning rate set for the optimizer was $10^{-5}$.

From the 400 cases in the training dataset, 100 were used for cross-validation while fitting the model. Thus, the model prevented overfitting and reduce prediction errors. As said before, the function loss used was the MSE, and the training required 15000 epochs until a platoon was reached.

#### Momemtum source from the CFD simulation of a real propeller

Finally, following the idea of using the CFD simulations as a ’virtual laboratory’, a real propeller has been simulated to evaluate the ability of the trained NN to propose momentum sources that can properly emulate its global effect. For the selection of the geometry, the aim was to find a model similar to the ones used in wastewater treatment tanks, i.e., a propeller with 3 blades. A CAD file for a three blade propeller based on the Dynaman-DYP-1017 model was rescaled to match the dimensions of the MS simulations and was inserted in a domain of the same dimensions that served for the remaining CFD cases (see Figure 3 of the supplemental information).

A transient isothermal simulation was configured with the propeller inside a cylinder subgion (Sliding Mesh) and was set to rotate at 60 rpm. The SST model for turbulence to describe adequately the flow near the walls.

A series of points have been extracted after performing a spatial average at the same locations that feed the NN and are shown in Figure 2 of the supplemental information. The trained NN provided momentum source terms according to these inputs which, in turn, have been implemented in an MS simulation. The profiles from this last simulation are then compared with the ones from the transient SM simulation.

## Supplemental information

Document S1. Figures S1‒S4
