# Supplementary material for: On the use of deep learning and computational fluid dynamics for the estimation of uniform momentum source components of propellers
Source: iScience. 2023 Oct 27;26(12):108297. doi: 10.1016/j.isci.2023.108297 (PMC10663753; doi:10.1016/j.isci.2023.108297)
Supplement: Document S1. Figures S1‒S4 [file mmc1.pdf]

iScience, Volume *26*

Supplemental information

On the use of deep learning and computational fluid dynamics for the estimation of uniform momentum source components of propellers

Raúl Martínez-Cuenca, Jaume Luis-Gómez, Sergio Iserte, and Sergio Chiva

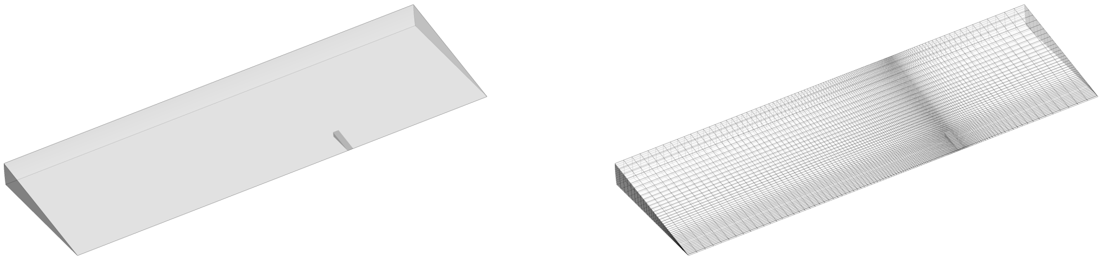

(a) (b)

Figure S1: Geometry (a) and mesh (b) used for the CFD simulations, related to STAR Methods.

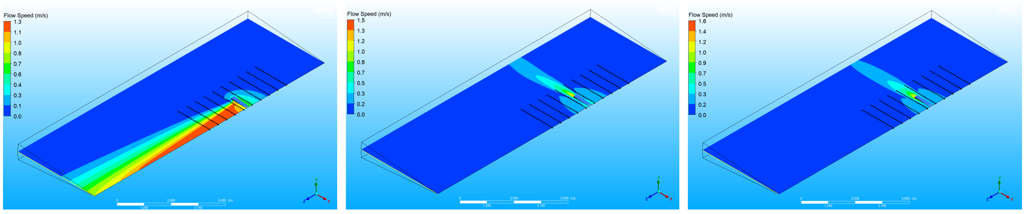

(a) (b) (c)

Figure S2: Resulting velocity fields for three CFD cases: a) $\mathbf{M} = 4000\ \mathbf{u_z}$, b) $\mathbf{M} = 4000\ \mathbf{u_r}$, and c) $\mathbf{M} = 4000\ \mathbf{u}_\theta$. The locations for the velocity samples are marked as dots, related to STAR Methods.

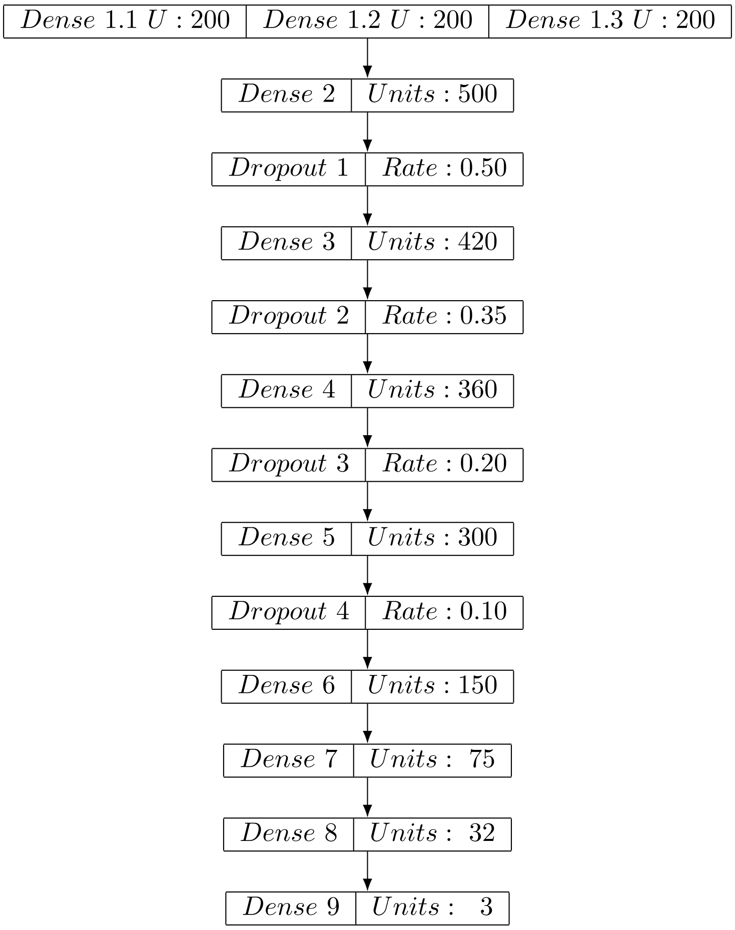

Figure S3: Neural network architecture, related to STAR Methods.

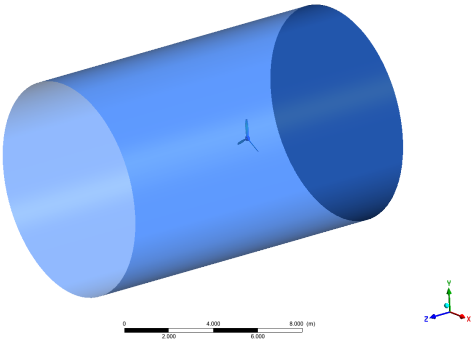

Figure S4: Snapshot from ANSYS showing the complete geometry of the case with the impeller at the same location as the momentum source subdomain, related to STAR Methods.
